# From tin at home: the other side of the coin of the breastfeeding

**DOI:** 10.1186/1824-7288-41-S1-A41

**Published:** 2015-09-24

**Authors:** Chiara Selmi

**Affiliations:** 1 USL 4, Prato, 59100, Italy

## 

The promotion of breastfeeding and the monitoring of the prevalence of breastfeeding at discharge of the newborn is one of the priorities of the health plan's integrated ToscanaRegion [1]. Since 2004, it implemented various measures to promote breastfeeding and has been involved in various investigations: “Birth Path” on a sample of 1,657 women who had given birth year previous [2], “Being a mother informed” on 2,324 pregnant women in birth centers Toscani, your raving two to three months after the childbirth[3]; and finally a survey conducted in 2010 in which 5,885 questionnaires were administered to parents who accompanied the children to make the first or second dose of vaccine required, in 44 vaccination centers AUSL championships in the region. Since 2008 data on the prevalence of breastfeeding are found on the Certificate of Assistance at Childbirth (Cedap) compiled before discharge. This will get annual data on the entire population. Comparing data obtained in the investigation there was an increase of exclusive breastfeeding at discharge 66% in 2001 to 76% in 2010. The increase was confirmed in 2012 by Cedap service where, it should be noted, a proportion of exclusive breastfeeding by 85 % with higher percentages to ‘80 % in the Baby-friendly Hospital of Tuscany.

The practice of breastfeeding exclusively decreases greatly from the earliest months of life. In fact, in the survey of 2010, children breastfed exclusively were 57.5%, 17.8% is nursed in a complementary and 24.7% were not breastfed. It would seem that mothers have the perception of having “less milk” or feel disoriented by the sudden increase in demand from nurse, often it is the so-called “crisis of the third month” when the child begins to show more interest to the environment exterior that is highly stimulating; or a “growth spurt” due to the increased demand of the growing child and needs to encourage more breast attaching more often. His most distractibility breast is interpreted by the mother as a reluctance to suckle the breast and this affects the maternal perception of not having enough milk. 

## References

[B1] Piano socio-sanitario integrato 2012-2015 http://www.ars.toscana.it/files/aree_intervento/salute_di_bambini/allattamento_SIDS/toscana_pssir_2012_2015.pdf

[B2] Casotto V Cuttini M Genovese I Il Percorso Nascita: risultati dello studio in Toscana Documenti dell'Agenzia Regionale di Sanità della Toscana n. 12 2005 Marzo

[B3] Puglia M Casotto V Rusconi F Essere Mamma Informata: allattamento al seno e SIDS Documenti dell'Agenzia Regionale di Sanità della Toscana n. 25 2007 Gennaio

